# Monitoring the Corrosion of Steel in Concrete Exposed to a Marine Environment

**DOI:** 10.3390/ma13020407

**Published:** 2020-01-15

**Authors:** Nina Gartner, Tadeja Kosec, Andraž Legat

**Affiliations:** Slovenian National Building and Civil Engineering Institute, Dimičeva ulica 12, 1000 Ljubljana, Slovenia; tadeja.kosec@zag.si (T.K.); andraz.legat@zag.si (A.L.)

**Keywords:** corrosion in concrete, long-term corrosion monitoring, real environment exposure, corrosion sensors, electrical resistance (ER) probes, coupled-multi-electrodes (CME), macrocell corrosion, stainless steel reinforcement

## Abstract

Reinforced concrete structures require continuous monitoring and maintenance to prevent corrosion of the carbon steel reinforcement. In this work, concrete columns with carbon and stainless steel reinforcements were exposed to a real marine environment. In order to monitor the corrosion processes, two types of corrosion probes were embedded in these columns at different height levels. The results from the monitoring of the probes were compared to the actual corrosion damage in the different exposure zones. Electrical resistance (ER) probes and coupled multi-electrodes (CMEs) were shown to be promising methods for long-term corrosion monitoring in concrete. Correlations between the different exposure zones and the corrosion processes of the steel in the concrete were found. Macrocell corrosion properties and the distribution of the separated anodic/cathodic places on the steel in chloride-contaminated concrete were addressed as two of the key issues for understanding the corrosion mechanisms in such environments. The specific advantages and limitations of the tested measuring techniques for long-term corrosion monitoring were also indicated. The results of the measurements and the corrosion damage evaluation clearly confirmed that the tested stainless steels (AISI 304 and AISI 304L) in a chloride-contaminated environment behave significantly better than ordinary carbon steel, with corrosion rates from 110× to 9500× lower in the most severe (tidal) exposure conditions.

## 1. Introduction

Reinforced concrete structures are not as long-lasting as was generally considered up until the 1970s [1,2]. Depassivation of the carbon steel that is normally used to reinforce the concrete structures occurs mainly due to corrosion processes, resulting in a loss of cross-section and a significantly reduced service life (or even the collapse) of the reinforced structure [3]. The economic, ecological, and safety aspects of this led to the beginning of intensive research to understand the mechanisms of corrosion in concrete, and to find new technologies and materials, with the aim of improving the durability of reinforced concrete structures [1,4].

The main two initiators of corrosion in concrete are carbonation and the presence of chloride ions [2,3,5]. However, the experimental results show that the carbonation rate in medium- and high-quality concrete is much lower than the chloride penetration rate, i.e., the carbonation mechanism is of little significance in a chloride-contaminated environment [6]. A lot of studies defining the chloride threshold level (CTL, i.e., the critical chloride concentration causing corrosion initiation) values in concrete have been published and the reported results scatter over more than two orders of magnitude when expressed as the total chloride content by cement weight [3,7]. Angst et al. [8] conducted research indicating that free Cl^−^ activity varies considerably, even at a constant depth (the local chloride concentration can deviate from the average chloride concentration by ±20% to ±70% [9,10]); therefore, the CTL might be over- or underestimated. However, the mentioned research [9] also concluded that the corrosion of steel in concrete does not necessarily initiate where the highest chloride concentration is. In fact, the most significant parameter influencing corrosion performance is the steel/concrete interface, i.e., the steel and the concrete micro- and macroscopic characteristics at the interface, which can influence the local susceptibility of the reinforcement to corrosion initiation [11,12].

When steel contains more than 10.5% of chromium and a maximum of 1.2% of carbon, with or without other alloying elements, it is considered to be stainless [13]. A stainless steel reinforcement can have up to a 10 times higher critical CTL than carbon steel [2,14] and is considered as one of the best corrosion protection for concrete structures that are exposed in a chloride environment [15]. It is mostly austenitic (e.g., AISI 304 and 316) and/or some duplex [16,17,18] stainless steels that have been subject to corrosion investigations. Regardless of the studied environment or the methods used, the conclusions are similar: the corrosion resistance of stainless steel reinforcements in a concrete environment is significantly better than ordinary carbon steel reinforcements [17,18,19]. It was also concluded that the direct (galvanic) contact of stainless and carbon steel reinforcing bars does not increase the corrosion risk for carbon steel [18,20,21], which means it is possible to use stainless steel only for the critical parts of concrete structures and for the rehabilitation of damaged parts.

Various electrochemical methods (potential mapping, galvanostatic pulse, electrochemical impedance spectroscopy, potentiodynamic polarization measurements) are used for the corrosion monitoring of steel in concrete [22,23,24,25,26], but their interpretation can be difficult and, in some cases, unreliable. The specific feature of corrosion in concrete is that the anodic and cathodic sites are spatially localized due to the porous structure of the concrete [5], while the porosity of the concrete also affects the transport of the electrolyte and oxygen, determining the dynamics of the corrosion processes [27]. Therefore, certain new or upgraded methods were developed, enabling the continuous monitoring of corrosion [28,29,30,31]. One specific type of embedded sensor is the electrical resistance (ER) probe, which measures the thickness reduction due to corrosion [28]. Specific applications of these probes in concrete [30,32] and bentonite [33] have proved that they can accurately determine the cumulative corrosion damage to a metal in porous materials. Coupled multi-electrode arrays (CMEAs) are somehow an upgrade of the electrochemical noise (EN) technique [34,35]. A CMEA allows measurements of partial corrosion currents, and therefore the monitoring of the corrosion pattern and the rate over time are possible [36]. Despite the wide-ranging capabilities of this technique, due to the complexity of the measuring setup, a CMEA has only been used a few times for corrosion in porous materials such as concrete [28,30] and bentonite [37]. A specific problem in measuring and interpreting steel corrosion in concrete is the fairly large interval of dimensions, which are involved in corrosion processes. In one part, the distribution of anodic and cathodic sites at the micro-scale is important, whereas on real concrete structures, this distribution could range up to a meter [38,39]. This wide range of dimensions should also be considered for reliable corrosion monitoring of steel in concrete [40,41].

The aim of this paper was to evaluate methods for the long-term corrosion monitoring of different steel reinforcements in a real marine environment and to compare the corrosion behavior of carbon and selected stainless steels. Concrete columns were reinforced with commercially available ribbed reinforcement bars made of carbon steel and austenitic AISI 304 and AISI 304L stainless steels and exposed on the Adriatic coast for 52 months. In order to evaluate the corrosion behavior at specific exposure zones, various corrosion probes (ER, coupled multi-electrodes (CMEs)) were placed at different levels of these columns. These results were compared with the actual corrosion damage to the rebars and the probes after the exposure. With respect to the limited availability of results on long-term corrosion tests in a real environment and the time-consuming aspect of this kind of investigation, the advantages, limitations, and also lessons learned with respect to the tested measuring techniques were indicated. In addition, the results obtained on the stainless steels (AISI 304, AISI 304L) were compared to the results measured on commonly used carbon steel.

## 2. Materials and Methods

### 2.1. Materials, Specimens, and Exposure Conditions

**Figure 1:**
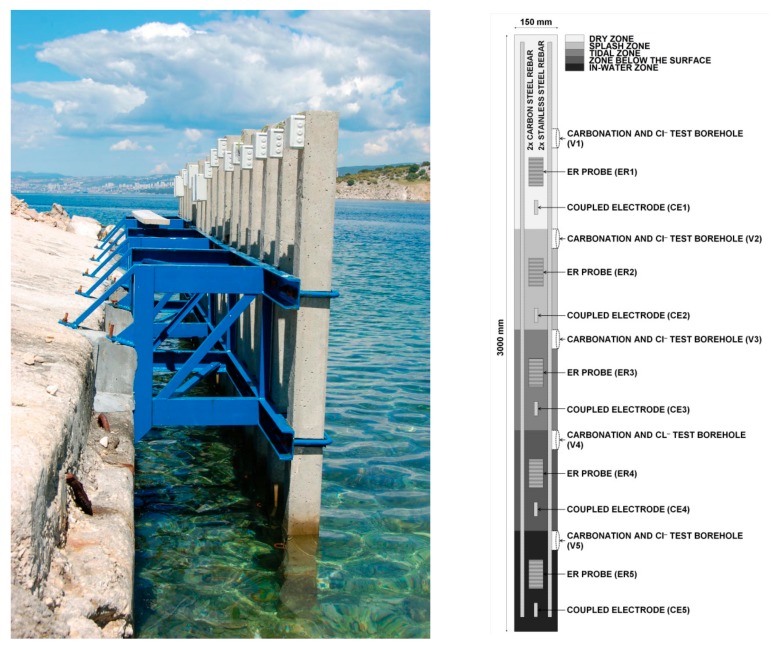
(**a**) Photograph and (**b**) schematic of a concrete column, placed vertically into the seawater, with the location of the steel reinforcement bars, electrical resistance (ER) probes, and coupled multi-electrodes (CMEs) at different heights (different exposure zones).

Large-scale concrete specimens (Figure 1) were prepared and exposed to a marine environment on the north Adriatic coast under the Krk Bridge (Croatia) for 52 months (4.3 years). The location of the test site was chosen because of its extreme environment, i.e., strong winds (bora and sirocco) and the high salinity of the water (3.5%) [42].

Altogether, 18 concrete columns, 3 m high and 15 cm × 15 cm wide, were prepared and exposed; however, only 4 columns (C10, C11, C12, C16) were equipped with ER and coupled multi-electrode (CME) sensors for long-term corrosion monitoring (Table 1, Figure 1) and are therefore presented in this paper. The bottom part of the columns was placed vertically into the sea, enabling corrosion monitoring in five different exposure zones: in-water zone (0 to 0.5 m from the bottom), zone just below the sea surface (0.5 m to 1 m from the bottom), tidal zone (1 m to 1.5 m from the bottom), splash zone (1.5 m to 2 m from the bottom), and dry zone (2 m to 3 m from the bottom). All the concrete columns were prepared using a concrete mixture with CEM I 42.5 cement (Salonit Anhovo, Slovenia) and a w/c ratio of 0.75, with no chemical additives. A high w/c ratio was used in order to accelerate the experiment and enable a qualitative comparison between the corrosion resistance of ordinary carbon steel and corrosion-resistant steel. The columns were cured for 28 days and were not subjected to accelerated carbonation before the exposure. The concrete cover thickness above the reinforcing steel bars, ER probes, and coupled multi-electrodes was 20 mm.

Each column was reinforced with two bars made of stainless steel and two bars made of carbon steel (Table 1, Table 2), with a 14 mm cross-section diameter, which were not physically connected. All the reinforcing bars were visually examined after the end of exposure.

In the selected four columns, five electrical resistance (ER) probes [34] made of stainless or carbon steel were installed in separate zones of exposure. ER probes suitable for concrete application were developed and patented at Slovenian National Building and Civil Engineering Institute in 2007 [44]. The carbon steel ER probes were produced from thin, 300 ± 10 µm strips of low-carbon steel grade 1.0038 (S235JR, according to the European Standard EN 10025-2 [43]) in the normalized condition. The stainless steel ER probes were made of grade AISI 304 (1.4301, according to the European Standard EN 10088-1 [13]) austenitic stainless steel plates, with a thickness of 100 µm ± 1 µm. The chemical compositions and the physical properties of both ER probes were very similar to the properties of the representative grade of reinforcing bars (Table 2). Two branches of exposed resistor element represented the corroding part of the probe, with an area of 4 cm^2^ for the carbon steel and 6.4 cm^2^ for the stainless steel ER probe. The other two branches of both ER probe types were protected with glass fiber and an epoxy coating, serving as a compensating (non-corroding) part of the ER probe.

**Figure 2:**
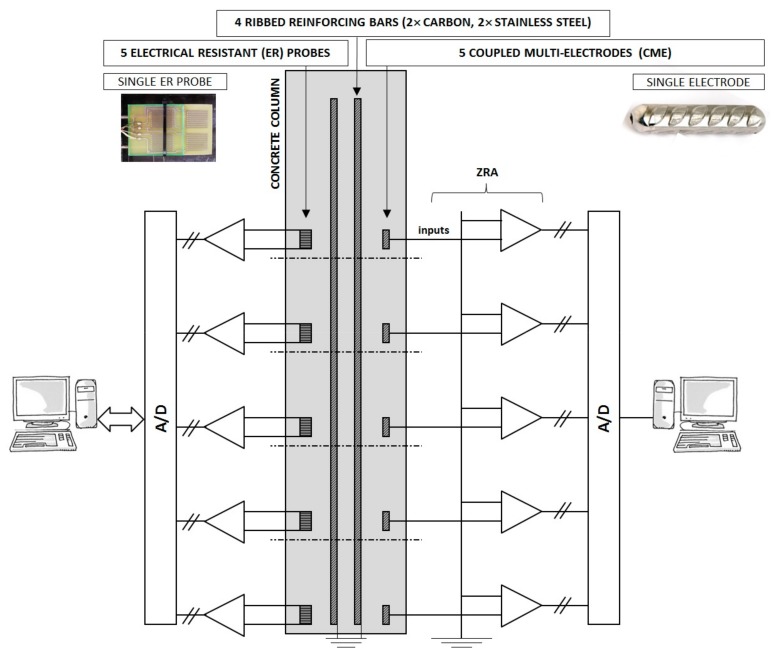
Schematic presentation of five individually measured electrical resistance (ER) probes and five coupled multi-electrodes connected through the Zero Resistance Ammeter (ZRA), with a photograph of a single ER probe and single electrode.

In addition to the reinforcing steel bars and ER probes, the same four columns were equipped with five special coupled multi-electrodes (Figure 2) made of carbon or stainless steel reinforcement (nominal diameter, 14 mm; exposed external surface, 25 cm^2^), placed with one in each zone of exposure (Figure 1, Table 1), and electrically connected through Zero Resistance Ammeters (ZRAs, ZAG, Ljubljana, Slovenia).

### 2.2. Methods

An ER probe is a sort of instrumented corrosion coupon: the thickness of the metal conductor is reduced due to the corrosion, while the resulting increase in its resistance is measured. Therefore, it is not an electrochemical but a physical method. Based on its performance characteristics, it is possible to determine the cumulative corrosion damage, as well as the corrosion rate in specific periods. The exact operating principle for ER probes was described in our previous papers [28,30,34,45]. The voltage drop over the entire ER probe (U) and the voltage drop between two branches (ΔU) were measured with a Portable Current Source SCS-100 (ZAG) at a 50 mA applied current and a Fluke 289 high-impedance voltmeter (Fluke Corporation) with an accuracy of 0.025%. Measurements of the thickness change had a resolution of less than 0.1 µm. The Wheatstone bridge configuration ensures the elimination (self-compensation) of temperature fluctuations. The configuration of ER probes in individual columns is presented in Figure 2.

Measurements of partial currents on CMEs is an upgrade of classic electrochemical noise (EN) measurements [30], and in its micro-scale arrangement is also referred to as the coupled multi-electrode array (CMEA) technique [36]. It measures separate anodic and cathodic corrosion currents in a chosen geometrical configuration, so the monitoring of the spatio-temporal evolution of the corrosion processes is possible. Five electrodes in one column were virtually short-circuited through the Zero Resistance Ammeter (ZRA) and thus acted as a single electrode surface, representing one reinforcing bar. The ZRAs were connected to a 16-bit analog-to-digital (A/D) converter (Figure 2). Each separate measurement was continuously carried out at a sampling frequency of 10 s^−1^ for a duration of 1–2 h.

Both techniques, ER probes and the CMEs, monitored the corrosion processes at the same five zones of exposure: dry, splash, tidal, below the surface, and in-water zone. Due to their positions, the purpose of the ER probes and the CMEs was to mimic the corrosion behavior of the reinforcing bar inside the concrete column in order to investigate primarily the macrocell corrosion processes.

After 52 months of exposure, the four columns (C10, C11, C12, and C16) were transported to the laboratory facilities for the final examinations. First, there was a visual inspection of the embedded carbon and stainless steel reinforcing bars and the coupled multi-electrodes after cleaning with HCl (conc.):H_2_O = 50:50 (vol.%) + 3 g/L urotropine solution. The carbonation depth was determined by spraying a phenolphthalein solution on freshly split cylinders (Φ 7 cm) cut from all five exposure zones of the column. The tests were performed according to the European Standard EN 14630 [46]. The concentration of chlorides at different concrete depths was determined in all five exposure zones of the column. The tests were performed according to the European Standard EN 14629 (Procedure A—Volhard method) [47].

## 3. Results

### 3.1. Corrosion Monitoring with ER Probes and CMEs

**Figure 3:**
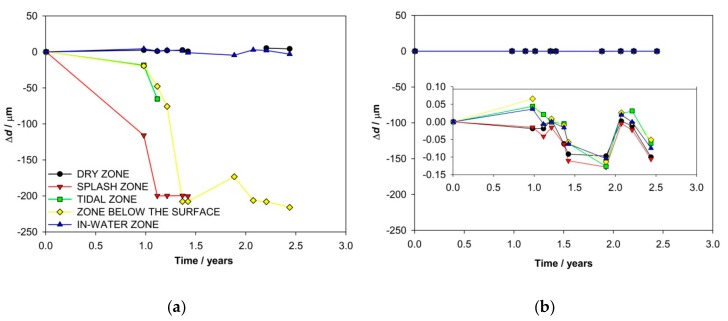
Thickness reduction of ER probes in different zones of exposure made of (**a**) carbon steel (column C11) and (**b**) stainless steel (column 10).

The ER probes’ thickness reduction Δd was measured in the five zones of exposure for the four concrete columns. Two columns were equipped with carbon steel and two with stainless steel ER probes. The thickness reduction on the carbon steel ER probes in the individual zones of exposure is almost identical in both equipped columns (C11 and C12); therefore, only the results from one column are presented (Figure 3a). The initiation of the corrosion in the splash, the tidal, and the zone below the surface was detected very early, after one year of exposure. The initiation was observed from the increased thickness reduction (Δd), which is directly related to the corrosion rate (faster thickness reduction, higher corrosion rate). The corrosion rates in the second year of the exposure increased from roughly 100 μm/year to a few hundred μm/year. According to our previous research [28,30], such fast measured thickness reduction indicates localized corrosion damage. Due to this, the estimation of the general corrosion rate can be misleading. No evident thickness reduction was measured on the ER probe exposed in the in-water zone. On the other hand, even a slow increase of the thickness was observed on the ER probe exposed in the dry zone. This can be explained by the unwanted corrosion of the compensating (protected) resistor elements, which increased its resistance. Unfortunately, an exact examination of the corrosion damage to these probes could not be performed, since it was not possible to dismantle the corroded ER probes from the concrete after the end of the exposure.

ER probes made of AISI 304 stainless steel were embedded in each zone of exposure for the two concrete columns (C10 and C16). The results measured in either column showed no corrosion activities for all 1569 days of exposure (Figure 3b). The short-term fluctuations of thickness (up to 0.15 μm) are below the detection limit of the measuring system and can be attributed to instrumental noise.

**Figure 4:**
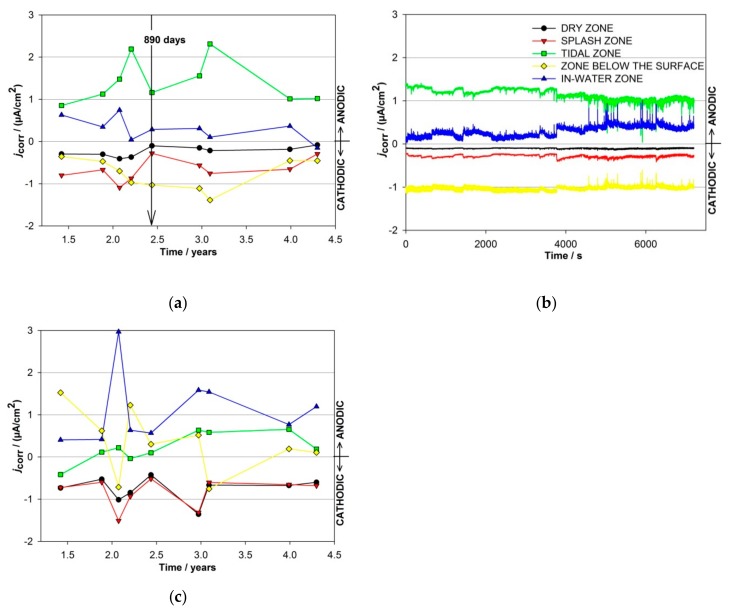
(**a**) Average values of the partial currents and (**b**) individual measurements of the partial currents after 890 days of exposure on carbon steel-coupled multi-electrodes exposed to different zones of exposure in column C11; (**c**) average values of the partial currents on carbon steel-coupled multi-electrodes exposed to different zones of exposure in column C12.

The anodic and cathodic currents were also measured in the different exposure zones by coupled multi-electrodes, which were placed in four concrete columns, one in each zone of exposure. During each 1–2-h-long measurement, current fluctuations were monitored on the individual electrodes (Figure 4b). At the time of one measurement, the currents on the individual electrode never changed between the anodic and cathodic positions. In order to follow the shifts of the anodic and cathodic sites between the different zones of exposure during the entire time of exposure, each measurement was averaged and presented as a function of time (Figure 4a).

The arrow in Figure 4a represents the average values of a 2 h measurement on the 890th day of exposure (Figure 4b). Certain similarities were observed in the current responses measured in both columns equipped with carbon steel-coupled multi-electrodes (C11 and C12). The average partial currents measured in columns C11 and C12 are presented in Figure 4a,c, respectively. Only the cathodic currents were measured in the dry and splash zones of both columns with the carbon steel-coupled multi-electrodes. The highest anodic currents in column C11 were measured in the tidal zone (2.3 µA/cm^2^), while the highest anodic currents in column C12 (3 µA/cm^2^) were measured in the in-water zone. These corrosion currents correspond to approximately 25 µm/year and 30 µm/year. The zone below the surface was the most cathodic in column C11 (−1.4 μA/cm^2^), while the currents shifted between cathodic and anodic in another column (up to 1.5 μA/cm^2^ and down to −0.7 μA/cm^2^). Low cathodic responses were measured in the dry area of column C11 (down to −0.4 µA/cm^2^), while the amplitudes of the cathodic currents measured in the dry zone of column C12 were slightly higher, i.e., −1.4 µA/cm^2^.

**Figure 5:**
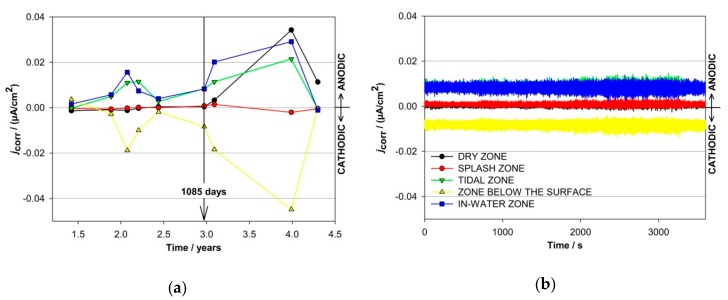
(**a**) Average values of the partial currents and (**b**) individual measurements of the partial currents after 1085 days of exposure on stainless steel-coupled multi-electrodes exposed to different zones of exposure in column C10.

Partial currents were also measured on five coupled multi-electrodes made of stainless steel, placed in five exposure zones of the concrete columns C10 and C16. The results in both columns showed very low values for the measured currents, i.e., no evident corrosion activities were observed on the electrodes made of AISI 304 embedded in column C10 (Figure 5).

In column C16 (electrodes made of AISI 304L) the highest measured anodic current was slightly higher than in column C10, but still relatively low (j_corr_ < 0.7 μA /cm^2^, corresponding to ν_corr_ ~7 μm/year, measured in the in-water zone electrode), while the average anodic currents were again negligible (corresponding to ν_corr_ ~0.01‒2 μm/year, higher at the beginning of the exposure).

**Figure 6:**
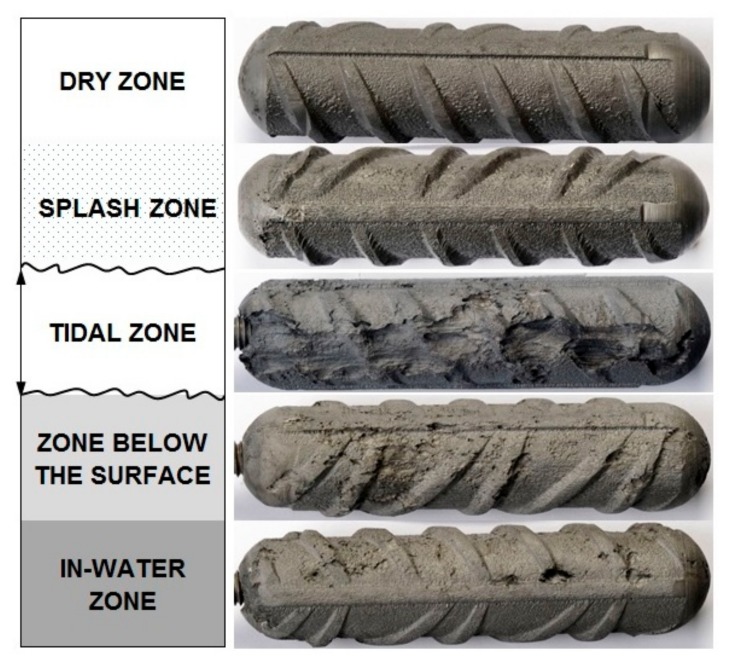
Photographs of the corrosion damage on the carbon steel-coupled multi-electrodes after extraction from the column C11.

A visual analysis indicated that the carbon steel-coupled multi-electrodes (CMEs) embedded in column C11 suffered corrosion in all the zones of exposure (Figure 6). The extent of the visual corrosion damage on these individual electrodes mostly correlates with the measured partial anodic currents (Figure 4), but not entirely. The visual examination confirmed that the highest corrosion damage was generated on the electrode exposed in the tidal zone, whereas slightly less corrosion damage was found on the electrode exposed in the in-water zone. These correspond to the anodic currents measured in both zones. The electrodes exposed to the dry and splash zones are the least damaged, which is in agreement with the predominant cathodic currents. However, the visual examination indicated relatively severe corrosion damage on the electrode exposed in the zone below the surface, which does not correspond to the measured cathodic currents. One of the reasons for this inconsistency is that the monitoring was not continuous, and there is no information about how the currents were distributed between the separate measurements. The second possible reason is the simultaneous presence of anodic and cathodic corrosion processes at the same electrode, so only the difference between the processes was measured. Apparently, the use of a CME system requires an optimal surface area of the individual electrode, which should be small enough to enforce the special division of anodic and cathodic sites and large enough to minimize the local steel-concrete non-uniformities.

The visual examination of the stainless steel CMEs showed no evident corrosion damage and corresponds to very low measured coupling currents.

### 3.2. Visual Examination of Reinforcing Steel Bars

The visual analysis of the carbon steel reinforcing bars embedded in column C11 after the 52 months of exposure also indicated different extents of corrosion damage in all the zones. Due to the large size of the exposed reinforcing bars, the entire length could not be visually presented in this paper. The corrosion is more-or-less uniform throughout the entire length of the bar. However, there are a few spots where the corrosion is deeper. A basic evaluation of the corrosion damage on the rebar is presented in Table 3.

The most severe and the deepest corrosion damage (i.e., 2.4 mm, which represents 17% of the reinforcement diameter) were estimated in the dry zone (Table 3). This result is not consistent with the cathodic corrosion currents measured on the coupled multi-electrodes in the same zone and is also not in agreement with the visual examination of this electrode (Figure 6).

**Figure 7:**
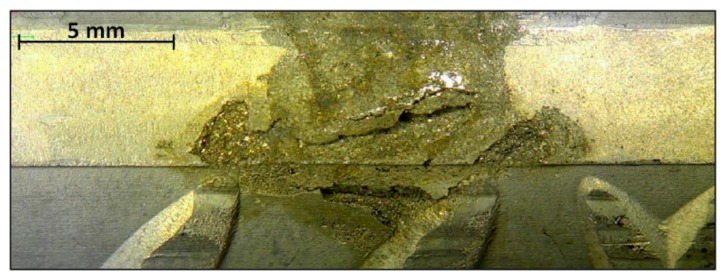
Optical image of the most severe corrosion damage found on a stainless steel reinforcement in concrete column C11 (located in the dry zone).

In contrast to the carbon steel rebars, the stainless steel reinforcing bars (AISI 304) in the same column C11 were practically undamaged. However, two corrosion spots were found, each on a separate bar, but both exposed to the dry zone. The damaged areas are not deep (below 500 μm), appearing on a very small area and representing the most severe damage found on the stainless steel (Figure 7). In both cases, it is assumed that the corrosion occurred at places where existing mechanical damage caused the corrosion initiation on the steel surface.

### 3.3. Carbonation Depth and Chloride Contamination

After the end of the exposure, destructive tests were performed on the concrete columns in order to determine the depth of carbonation and chloride concentration at different concrete depths and zones of exposure. The carbonation depth variation for the different zones of exposure was expected (not graphically presented): after the end of experiments no carbonation was found in the zone submerged in the water and the deepest carbonation (6 mm deep) was detected in the dry zone. Taking into account the 20 mm concrete cover, we can conclude that in the 52 months of exposure, carbonation did not reach the steel reinforcement and the embedded probes. Therefore, it can be concluded that the influence of the carbonation on the corrosion processes for the embedded steel was insignificant. However, it should be noted that the phenolphthalein test only shows pH values that are lower than 8.5.

**Figure 8:**
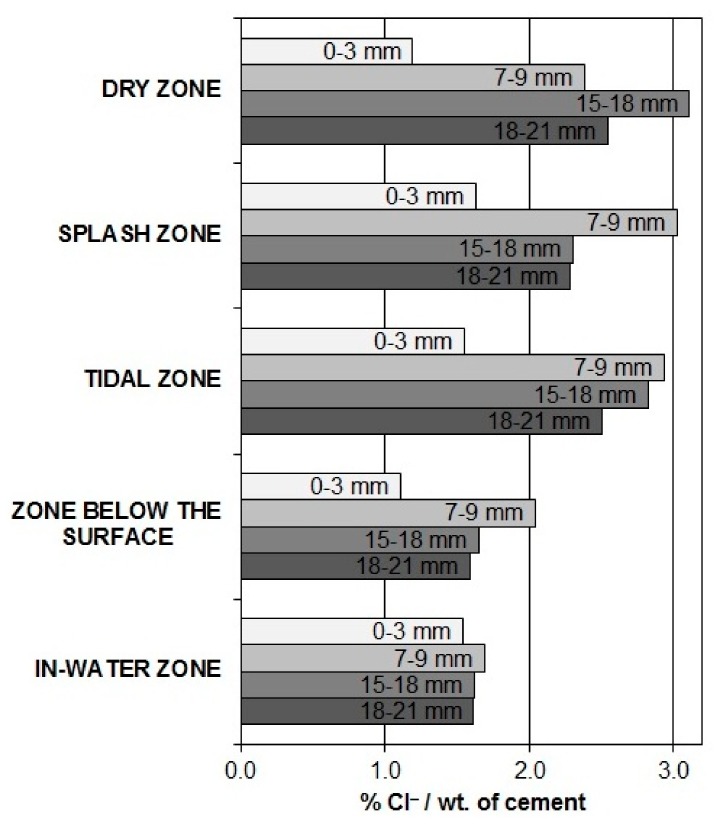
Total Cl^‒^ content in all the exposure zones at four different concrete depths.

In all the zones of exposure, the lowest contents of Cl^−^ (Figure 8) were found close to the surface of the concrete (0–3 mm deep), where the chlorides can be washed out. In general, the highest contents of Cl^−^ were detected at a concrete depth between 7 mm and 9 mm. At this depth, the Cl^−^ content is the highest in all the zones of exposure, except in the dry zone, where the highest Cl^−^ content was found to be deeper (between 15 mm and 18 mm). On average, the highest Cl^−^ content was found in the tidal zone, although the highest Cl^−^ content at the level of the steel reinforcement and probes was detected in the tidal zone and the dry zone. The splash zone followed with just a slightly lower percentage. These zones were exposed to the harshest environmental conditions (e.g., strong winds, heavy rain, constant wetting, and drying, etc.) that can penetrate chlorides deeper into the concrete [42]. The lowest Cl^−^ content in general and also in the depth of the steel reinforcement was measured just below the water surface and in the in-water zone, where the distribution of Cl^−^ at different depths is very uniform. This indicates very stationary conditions concerning the diffusion of chlorides through the concrete.

It can be seen that the distribution of Cl^−^ content at the level of the embedded steel and probes does not fully correspond to the distribution of the corrosion damage in the different zones of exposure. On the other hand, it is evident that in all the zones at the level of the reinforcement and the probes the concentration of Cl^−^ is relatively high.

## 4. Discussion

The main aim of our long-term research was to evaluate two relatively new measuring techniques for the corrosion monitoring of steel in concrete at different heights at the coast. It was believed that specific corrosion processes, which are related predominantly to macrocell corrosion due to individual exposure conditions at specific heights (in-water, below the water level, tide, splash, and dry area), could be detected. Namely, due to the different concentrations of anions and oxygen, a particular distribution of anodic and cathodic sites can be expected. The electrical conductivity between these sites due to the presence of water certainly plays a significant role in their distribution. It should be mentioned that due to the tide and splashes this distribution is changing. It was expected that the measuring techniques (ER probes and CMEs) could also provide some relevant information about the eventual corrosion initiation on stainless steel in concrete. It was found that a distinct part of the obtained results was in agreement with our expectations, but specific questions that need further discussion and investigations were also indicated.

The visual analysis of the carbon steel reinforcing bars embedded in columns revealed serious corrosion damage. The corrosion was more-or-less general throughout the entire length of the bars, with several spots where the corrosion is deeper. These spots were usually denser in the tide and splash zones, but they were also present in all the other zones. Actually, there were no distinct borders between the different areas. Further considerations in combination with the analysis of meteorological data revealed that specific characteristics of the individual exposure zones were significantly disturbed by occasional strong winds and high waves. Despite the fact that these events were infrequent, their effect on the corrosion conditions in the concrete was large, and conclusively the “dry” zone could not be considered as dry at all. This observation is also in agreement with the chloride concentrations at different levels, where there is no significant difference between the tide, the splash, and the “dry” zone. Consequently, the basic expectation that in the different exposure zones due to individual conditions specific electrochemical processes should be generated, was not fulfilled. For this reason, a comprehensive analysis of the results measured in the individual zones and their interpretation was also only partly possible.

The ER probes measure the metal’s thickness reduction due to corrosion, so it is not an electrochemical but a physical method. Based on its performance characteristics, it is possible to determine the cumulative corrosion damage, as well as the corrosion rate in specific periods. It was found in our previous research that the first part of the thickness reduction results predominantly reflects general corrosion, but afterward (more than 20% of the reduction) localized corrosion could also be detected [28,30,34]. The highest corrosion rates using the ER probes were measured in the splash and tidal zones, whereas the corrosion rates in the zone below the surface were slightly lower. Since localized corrosion rates on rebars are usually considerably higher than the general corrosion rate, it can be concluded that in the faster increase of corrosion rates obtained by the ER probes actually both types our corrosion were measured. Negligible corrosion rates were measured in the dry and in-water zones.

The ER probes were, however, not electrically connected one to another and therefore both parts of the corrosion process (anodic and cathodic) were localized on each individual ER probe. In this aspect, the use of CMEs seems more realistic, since all the electrodes were electrically connected together (acting as one reinforcing rebar), which enabled a spatio-temporal separation of the anodic and cathodic currents between the individual electrodes. The corrosion rates obtained by the CMEs were, however, evidently lower than those measured by the ER probes and also their pattern was different: anodic currents were measured on the electrodes in the tidal, below surface, and in-water zones; cathodic currents were measured on the electrodes in the dry and splash zones. The highest corrosion rates were detected in the tidal zone. Corrosion currents in the “dry” zone were expected to be zero, but obviously, a level of humidity ensured certain corrosion processes. Due to the deep corrosion damage on the electrodes placed in the dry and splash zones, it can be concluded that at both electrodes the anodic and cathodic events were simultaneously present, but predominantly the cathodic part of the processes was measured.

The advantage of the CMEs technique should be that besides the anodic current it also enables detection of the cathodic processes, which was proved in our previous micro-scale research using relatively small electrodes and short distances between them [30]. In this manner, the evolution of specific corrosion patterns was also monitored. It can be concluded, however, that in the presented case the surface area of the electrodes was too large and also the distance between them was too long. This ensured that the anodic and cathodic activities occurred simultaneously at the same electrode. Some indications about this problem were observed already during the exposure, but unfortunately, specific improvements to the monitoring system (embedding additional probes) were not possible. On the other hand, certain signals of the partial anodic and cathodic currents were measured, which suggests promising prospects of applying the CMEs technique also at macro-scales when the overall configuration of the electrodes is optimized.

Another problematic parameter that should be improved is the sampling rate: the technological level of our corrosion monitoring systems at the beginning of this long-term exposure was unable to measure the various parameters automatically with higher frequency. Progress in measuring electronics and the big-data analysis allowed us to implement a continuous monitoring system with wireless data acquisition in our new testing field.

## 5. Conclusions

Two methods (electrical resistance probes and coupled multi-electrodes) were implemented for the corrosion monitoring of reinforced concrete columns that were exposed in a marine environment for 52 months. These techniques were found to be promising for the corrosion monitoring of steel in reinforced concrete structures, although some open issues still exist. The following major observations can be reported:

- The use of electrical resistance (ER) probes was a reliable method for the detection of corrosion initiation and the propagation of general corrosion. Due to their geometry, the ER probes could also provide information about localized corrosion.
- It can be concluded that the ER probes in our coastal exposure measured predominantly the so-called self-corrosion, since they were not mutually nor connected to the vertical rebars. The measured corrosion rates below the water level were close to zero.
- Based on experiences from small-scale experiments [30] it was expected that measuring with coupled multi-electrodes (CMEs) should be a unique technique that makes it possible to measure the spatio-temporal evolution of the corrosion process of steel in concrete, predominantly the macrocell corrosion. However, at larger scales, as was the case in our coastal exposure, the interpretation of the results was not so definite. One possible explanation was that due to the relatively large distance between the neighboring electrodes and their large surface area, the measured currents reflected a combination of macrocell and self-corrosion processes on individual electrodes.
- The visual examination of ordinary carbon steel B 500B reinforcing bars demonstrated severe corrosion damages, which were also indicated by means of both the used measuring techniques. On the other hand, the tested stainless steel reinforcing bars (AISI 304 and AISI 304L) showed very good corrosion resistance with no evident corrosion damage found during the 4.3-year exposure time in a harsh marine environment. These observations were confirmed by the minor response of the ER probes and the very low currents measured by the CMEs.

The results provided positive information about the applicability of each implemented monitoring method. On the other hand, specific concerns about the initial installation deficiencies and measuring parameters were raised. Due to the severe and changing exposure conditions, the expected characteristics of the exposure zones (in-water, below the water level, tide, splash, and dry area) were not definite: high waves generated a relatively high oxygen concentration in the water zone, and extremely strong winds occasionally wetted and contaminated the “dry” zone with chlorides. Such non-stationary conditions made the infrequent sampling rate of the measurements by ER probes and CMEs even more problematic, and the evolution and spatial distribution of the corrosion processes were detected only to a certain degree.

It is clear that reliable corrosion experiments under natural conditions require fairly long exposure periods. The results provided information about the applicability of each used method, including specific concerns about the initial installation deficiencies. Due to the specificity of long-term real environment research, these deficiencies will be corrected in our future experiments, where a continuous monitoring system with wireless data acquisition will be implemented.

## Figures and Tables

**Table 1:** Type of steel reinforcement bars, ER probes, and CMEs, embedded in each concrete column.

Column	Reinforcing Steel Bars		ER Probes	Coupled Multi-Electrodes
	Carbon Steel Grade	Stainless Steel Grade		
C10	B500B	AISI 304 (1.4301)	AISI 304 (1.4301)	AISI 304 (1.4301)
C11	B500B	AISI 304 (1.4301)	S235JR (1.0038)	B500B
C12	B500B	AISI 304 (1.4301)	S235JR (1.0038)	B500B
C16	B500B	AISI 304L (1.4306)	AISI 304 (1.4301)	AISI 304L (1.4306)

**Table 2:** Chemical composition of the tested steel grades.

Steel Name [43]	C wt %	Si wt %	Mn wt %	P wt %	S wt %	N wt %	Cr wt %	Cu wt %	Mo wt %	Ni wt %
Carbon Steel										
B500B (1.0439)	0.19	0.10	0.62	0.020	0.022	0.005	0.07	0.04	<0.02	0.02
S235JR (1.0038)	0.056	0.003	0.15	0.017	0.015	0.014	0.01	0.05	0.007	0.02
Austenitic (Cr–Ni) Stainless Steel										
AISI 304 (1.4301)	0.058	0.42	1.46	0.019	<0.001	0.051	18.2	0.11	0.036	7.93
Austenitic (Cr–Ni, low-C) Stainless Steel										
AISI 304L (1.4306)	0.016	0.38	1.50	0.025	0.008	0.028	18.2	0.44	0.107	9.22

**Table 3:** Corrosion evaluation of the carbon steel rebar in concrete column C11.

	Diameter Reduction (μm)	Corrosion Rate *ν*_corr_ (μm/year)	Maximum Pit Depth (mm)	Maximum Pit-Corrosion Rate *ν*_corr-pit_ (μm/year)	HEAVILY Corroded Area (%)*	MILDLY Corroded Area (%)**
Dry zone (first 0.5 m)	46	11	1.2	270	0.1	2.9
Dry zone (next 0.5 m)	58	14	2.4	550	0.6	1.6
Splash zone	146	34	1.6	370	1.5	4.7
Tidal zone	58	13	1.7	390	0.1	3.5
Below surface	75	17	1.9	440	2.9	1.7
In-water zone	10	2	0.2	50	0	0.1

* Area of corrosion damages deeper than 1 mm; ** Area of corrosion damages shallower or equal to 1 mm.
